# The antigen CD300e drives T cell inflammation in adipose tissue and elicits an antibody response predictive of the insulin sensitivity recovery in obese patients

**DOI:** 10.1186/s12950-022-00318-7

**Published:** 2022-11-22

**Authors:** Sara Coletta, Elisabetta Trevellin, Marisa Benagiano, Jacopo Romagnoli, Chiara Della Bella, Mario Milco D’Elios, Roberto Vettor, Marina de Bernard

**Affiliations:** 1 Department of Biology, University of Padova, Padova, Italy; 2 Medical Clinic III, Department of Medicine (DIMED), University of Padova, Padova, Italy; 3 Department of Experimental and Clinical Medicine, University of Florence, Florence, Italy; 4 Department of Medicine and Translational Surgery, Catholic University, Rome, Italy; 5 Department of Molecular and Developmental Medicine, University of Siena, Siena, Italy

**Keywords:** Obesity, Inflammation, T cell response, Antibodies, Insulin resistance

## Abstract

Obesity and insulin resistance (IR), the key features of metabolic syndrome, are closely associated with a state of chronic, low-grade inflammation. Bariatric surgery leads to a considerable reduction in the adipose tissue mass and systemic inflammation along with a reduction of IR, with a whole-body metabolic improvement. However, a sizable portion of people experience an IR relapse within few years of remission.

Numerous studies have attempted to explore the best clinical predictors of the improvement of insulin sensitivity and the maintenance of glucose homeostasis after bariatric surgery, but no simple fasting blood test has been found to be effective in predicting the short and long-term beneficial effects on glycaemia.

With the present study, we investigated T-cell and antibody responses against CD300e, an antigen highly expressed in the adipose tissue of patients with obesity before the bariatric surgery-induced weight loss. We found both in fat tissue and in peripheral blood anti-CD300e-specific T helper 1 responses. Moreover, we evidenced in the sera of individuals with obesity an antibody response towards CD300e and revealed the existence of a significant correlation between the level of antibodies before surgery and the maintenance of glucose control after the intervention.

## Introduction

Obesity is a chronic relapsing progressive systemic disease with a multifactorial pathogenesis [1]. In patients with obesity and/or metabolic syndrome, adipose tissue (AT) is usually dysregulated because of a complex crosstalk between the metabolic system and the immune system. Fat accumulation associates with insulin resistance (IR), changes in circulating levels of adipocytokines, low-grade chronic inflammation, increased infiltration of immune cells, and increased risk for the development of type 2 diabetes (T2D). The treatment of obesity is very challenging and includes bariatric surgery. Beyond important weight loss, bariatric surgery leads to significant early improvement of hyperinsulinemia and IR, according to the evaluation by the homeostatic model assessment for insulin resistance (HOMA-IR) [2].

Plasma of individuals with obesity and IR contains autoantibodies specific for several autoantigens [3]. The immune receptor CD300e has been shown to be expressed by macrophages infiltrating the AT of patients with obesity [4] and a study performed on a cohort of 49 monozygotic twin pairs, discordant for body mass index, revealed that the mRNA encoding CD300e is significantly up regulated in AT of the heavier subjects than their co-twin [5].

This study aimed at i) investigating if CD300e elicits an antibody response in patients with obesity and if the antibody titer declines after surgery-induced weight loss, ii) verifying if the anti-CD300e titer before weight loss might be predictive for the improvement of insulin sensitivity and glucose metabolism, and iii) exploring CD300e-specific T cell-responses both in the peripheral blood and in adipose tissues.

## Material and methods

### Patients

A total of 48 patients with obesity and eligible for bariatric surgery (laparoscopic sleeve gastrectomy) were enrolled at the Center for the Study and the Integrated Treatment of Obesity of the University Hospital of Padova, according to the following criteria: age 18–65 years with BMI ≥ 40.0 kg/m^2^ or with BMI between 35.0 and 39.9 kg/m^2^ and co-morbidities. Blood samples were collected from patients before bariatric surgery-induced weight loss and from the same subjects at the time of abdominoplasty. Blood from 72 individuals of normal weight (age 18-65 years with BMI between 18.5 and 24.9 kg/m^2^) were included as control. Insulin resistance was evaluated using the homeostasis model assessment: HOMA- IR = Fasting glucose (mmol/L) x Fasting Insulin (μU/mL)/22.5.

### Quantification of α-CD300e antibodies in human sera

The level of anti-CD300e antibodies in sera was evaluated by a home-made ELISA. Briefly, serum was isolated from blood samples and diluted 1:100 before adding to wells of a 96-well plate previously coated with recombinant CD300e (5 ng/well, Abnova). Horseradish peroxidase–conjugated anti-human IgG antibody (Calbiochem) were added to each well, and colour was developed with TMB (Ebiosciences). Absorbance was read at 450 nm.

### Generation of T cell clones from peripheral blood and adipose tissue and analysis of the profile

We investigated the T cell responses of patients affected by obesity and of controls not affected by obesity. Following authorization of the local ethical committee and informed consent, T cells were isolated from peripheral blood samples of 7 patients with obesity and from visceral adipose tissue (AT) of 3 donors undergoing laparoscopic bariatric surgery. From peripheral blood, CD300-e-specific T-cell lines were induced as described [6, 7]. Peripheral T cells isolated from 3 individuals of normal weight were included as control. Activated T cells were isolated and expanded from AT specimens. Briefly, each AT sample was cultured for 7 days in RPMI 1640 medium supplemented with 10% FCS and human recombinant IL-2 (50 U/ml) to expand preferentially T cells activated in vivo. AT specimens were then disrupted, and T-cell blasts were cloned under limiting dilution conditions in round-bottomed microwell plates containing irradiated peripheral blood mononuclear cells (as feeder cells), PHA (0.5%) and IL-2 (20 U/ml) [7]. Surface marker analysis of T cell clones was performed by a flow cytometer FACS Canto (BD Biosciences, USA) with the Diva software for acquisition (Becton Dickinson).

Clones were screened for responsiveness to CD300e antigens by measuring [3H] thymidine uptake after 60 h of co-culture with irradiated autologous mononuclear cells in the presence of CD300e (2 μg/ml). At 16 h before harvesting, 0.5 μCi of [3H] thymidine was added, and radionuclide uptake was measured in a β-counter. The mitogenic index (MI) was calculated as the ratio between mean values of cpm obtained in stimulated cultures and those obtained in the presence of medium alone. MI > 5 was considered as positive. To assess the cytokine production of CD300e-specific clones on antigen stimulation, T cells were co-cultured for 48 h with irradiated autologous peripheral blood mononuclear cells in the absence or presence of CD300e (2 μg/ml). At the end of culture period, duplicate samples of each supernatant were assayed for IFN-γ, IL-17 and IL-4 by ELISA (R&D).

### Statistical analysis

All statistical analyses were carried out using GraphPad Prism software version 9.0. Non-parametric Mann–Whitney U-test and Kruskal–Wallis were carried out to assess differences between continuous variables with Bonferroni correction for multiple comparison when appropriate. Non-parametric correlations were measured using the Spearman rank correlation coefficient. Receiver operating characteristic (ROC) curve analysis was used to assess the predictive potential of the anti-CD300e antibody content in patients’ sera.

## Results and discussion

Based on the evidence that patients with both type 1 diabetes and T2D are seropositive for CD300e [8], a surface receptor, expressed by myeloid cells [9], we wondered whether antibodies anti-CD300e were increased in obese individuals which often suffer from glucose dysmetabolism.

**Fig. 1:**
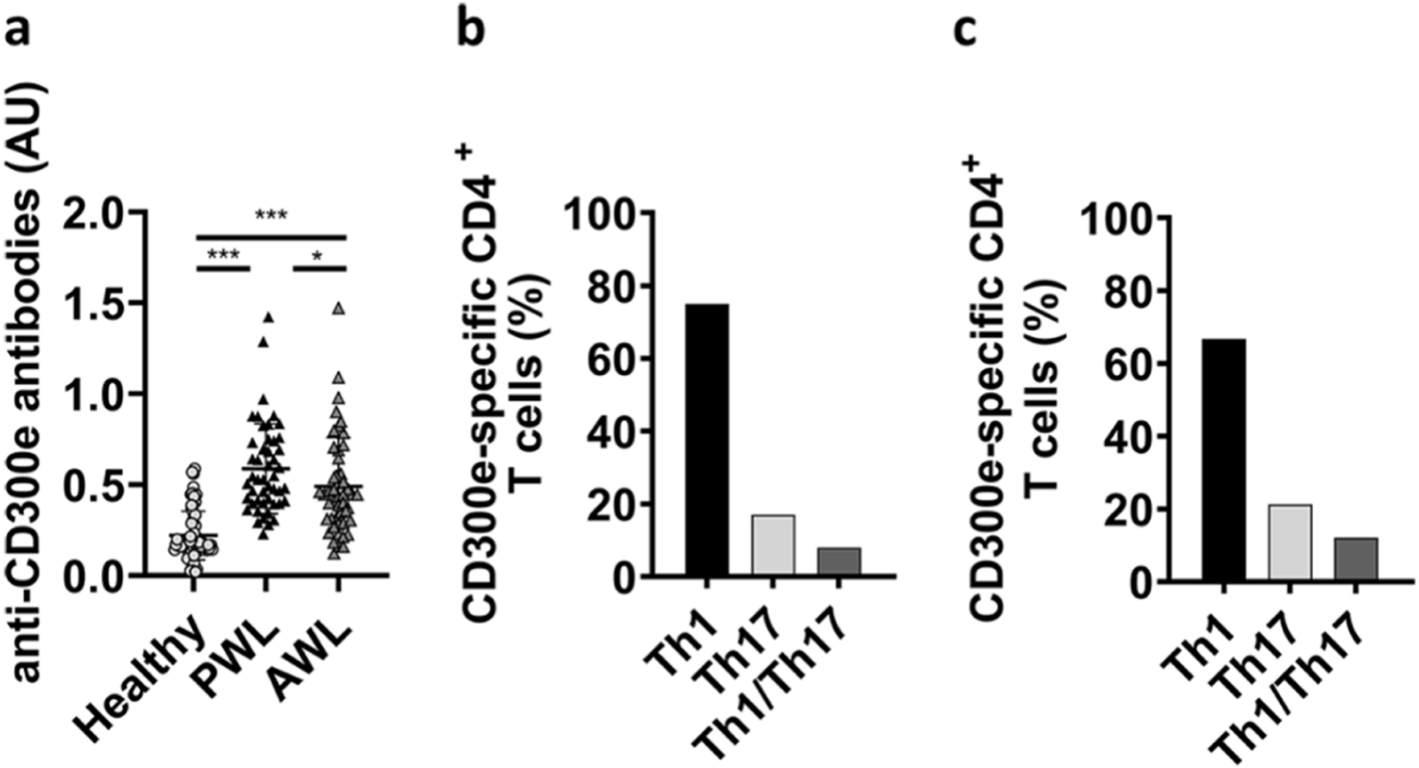
Obese patients are seropositive to CD300e and possess CD300e-specific CD4^+^ T cells with a pro-inflammatory profile. **a** Sera of 72 subjects of normal weight and of 48 patients with obesity, pre (PWL) and after (AWL) bariatric surgery-induced weight loss, were evaluated by ELISA against CD300e. Data are expressed as arbitrary units (AU). Significance was determined by Mann-Whitney U Test. *p ≤ 0.05; ***p ≤ 0.001. **b**-**c** Profile of CD300e-specific CD4^+^ T cells isolated from blood of 7 patient with obesity (**b**) or from AT (**c**) of 3 obese individuals

By comparing a cohort of 48 obese subjects with a cohort of 72 individuals of normal weight, we verified that sera from the former contained significantly more anti-CD300e antibodies than those from the latter. Notably, the comparison between the same patients before and after bariatric surgery-induced weight loss revealed that in 77% (37 patients out of 48) of patients the anti-CD300e antibody content declined (Fig. 1a).

CD4^+^ T cells are necessary as helpers to promote B cell antibody production. In accordance, we obtained 608 clones from peripheral blood of obese patients (81 clones, 89 clones, 86 clones, 93 clones, 87 clones, 75 clones, 97 clones respectively from each patient studied). 253 (41%) of the 608 clones derived from the peripheral blood of obese patients proliferated significantly in response to CD300e. Upon antigen stimulation, 189 clones (75%) displayed a Th1 profile in that they secreted primarily IFN-γ, 43 clones (17%), a Th17 profile characterized by the secretion of IL-17, and 21 clones (8%) produced both IFN-γ and IL-17 (Th1/Th17) (Fig. 1b). Interestingly, no CD300e-specific clones were obtained from individuals of normal weight. Moreover, we evidenced that the profile of CD4^+^ T-cells isolated from AT mirrored that of circulating T cells (Fig. 1c). AT-infiltrating in vivo activated T cells were expanded in vitro from 3 patients with obesity in a IL-2-conditioned medium, cloned, and studied for their phenotypic and functional profile. For each patient, CD4^+^ and CD8^+^ AT-derived T cell clones were screened in triplicate for proliferation in response to medium or CD300e. No CD8^+^ clones proliferated to CD300e. We obtained 161 clones from AT (50 clones, 62 clones, 49 clones respectively from each patient). 33/161 CD4^+^ clones were specific for CD300e: 22 of the 33 clones (66.7%) were Th1, 7 (21.2%) were Th17 and 4 (12.1%) were Th1/Th17.

Together, this evidence suggest that the CD300e-specific T cells in obese individuals mainly display the pro-inflammatory Th1 phenotype, which is the most represented in obesity and the fuel for AT and systemic inflammation and insulin resistance [10].

**Fig. 2:**
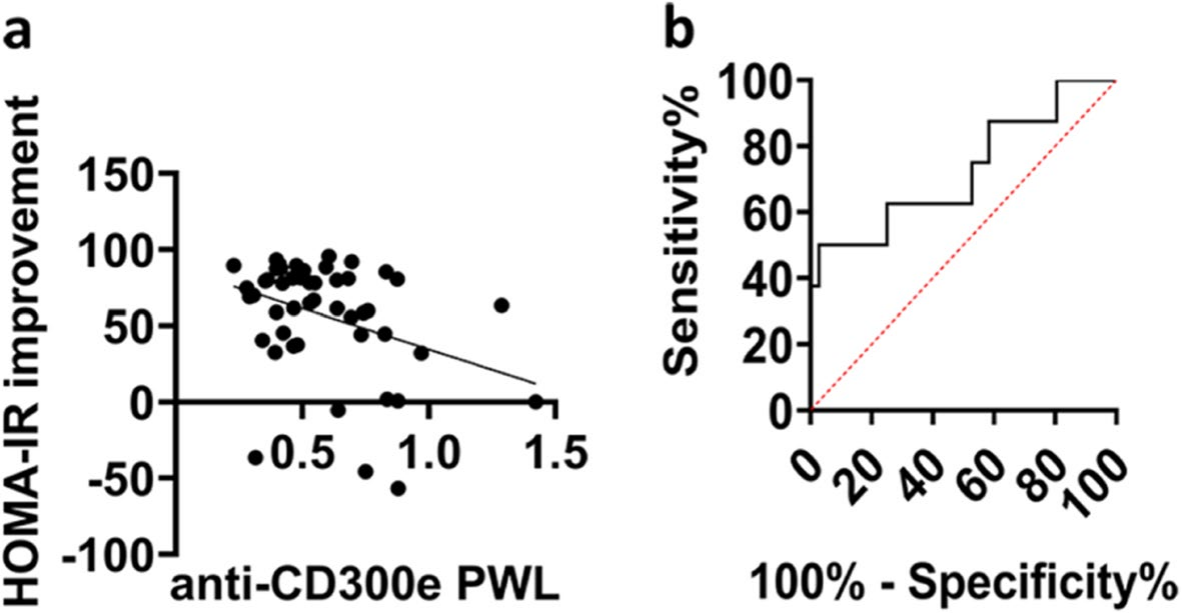
Anti-CD300e antibodies correlate with the improvement of insulin resistance in patients with obesity. **a** The presence of anti-CD300e antibodies in patients with obesity before weight loss inversely correlates with the amelioration of insulin resistance (expressed as % HOMA-IR improvement in AWL vs PWL) after bariatric surgery-induced weight loss. Non-parametric correlation was measured using the Spearman rank correlation coefficient (*R* = − 0.31; *P* = 0.02). **b** Receiver Operating Characteristic (ROC) curve analysis was used to determine the potential predictive role of serum anti-CD300e antibodies in the successful improvement of insulin sensitivity after bariatric surgery-induced weight loss (expressed as > 40% amelioration in HOMA-IR in AWL vs PWL). Cut-off > 0.64; Area under curve = 0.725; *p* = 0.047

Next, we calculated non-parametric correlations using the Spearman rank correlation coefficient between clinical characteristics of matched patients before and after weight loss (Table 1). Intriguingly, we found that the presence of anti-CD300e antibodies in sera of obese patients before weight loss inversely correlated with the improvement of HOMA-IR after weight loss (*R* = -0.31; *p* = 0.02) (Fig. 2a). These data suggested that the seroconversion to CD300e in obese patients may have a predictive role for the improvement of insulin sensitivity after bariatric surgery-induced weight loss. To corroborate this notion, we performed a ROC curve analysis to determine if the anti-CD300e antibody content in sera of patients before surgery could be useful to distinguish patients that improved their HOMA-IR (> 40%) from those who had a poorer amelioration of HOMA-IR (< 40%): We found that values lower than 0.64 arbitrary units (AU) can predict whether the insulin resistance status of the patient will successfully improve after weight loss (Area under curve = 0.752; *p* = 0.047) (Fig. 2b).

**Table 1:** Anthropometric and clinical characteristics of patients with obesity

	PWL	AWL	*p*
**Sex (M/F)**	20/28	–	
**AGE**	18-64	23-66	
**BMI**	35-68.3	23.9-48.4	< 0.0001
**Total cholesterol mg/dl (mean ± SD)**	187.17 ± 38.82	173.57 ± 33.54	0.0798
**Triglycerides mg/dl (mean ± SD)**	116.35 ± 56.25	80.93 ± 32.70	0.0003
**PCR mg/l (mean ± SD)**	6.88 ± 5.86	1.64 ± 1.99	< 0.0001
**HOMA-IR mg/dl (mean ± SD)**	5.19 ± 4.31	1.39 ± 0.946	< 0.0001
**Glucose mmol/l (mean ± SD)**	5.89 ± 1.73	4.54 ± 0.47	< 0.0001
**Insulin mU/l (mean ± SD)**	19.34 ± 14.77	6.83 ± 4.3	< 0.0001
**Leptina μg/l (mean ± SD)**	37.47 ± 15.93	11.11 ± 8.91	< 0.0001
**Anti-CD300e antibodies, AU (mean ± SD)**	0.59 ± 0.25	0.46 ± 0.27	0.0238

Continuous variables were analyzed with Mann-Whitney U Test if variables were not normally distributed. *P*-value below 0.05 was considered significant

Metabolic changes linked with a dysfunctional adipose organ in obesity leads to immune activation [11] with the shifting towards a chronic low-grade inflammatory state. The latter firstly involves the adipose organ, but gradually it expands at the systemic levels with a tremendous impact in the development of insulin resistance and related metabolic disease [12, 13]. AT also provides an environment for the secretion of IgG antibodies with anti-self-reactivity mostly due to the release of self-antigens.

Here we revealed that subjects with obesity produce antibodies against the immune receptor CD300e, in accordance with an increased expression of the protein in AT [5]. Conversely, the latter displays a low expression of CD300e following weight loss [14], and this is mirrored by the decline of the antibody titer. In our subjects with obesity the serum level of anti-CD300e antibodies before weight loss inversely correlated with the improvement of HOMA-IR after weight loss.

T cells play indispensable roles in tissue immunometabolism. An abnormal infiltration of CD4^+^ T cells was initially identified in human AT and significantly correlated with the body mass index [15]. CD4^+^ T cells were increased in AT of obese mice and produce higher amounts of IFN-γ than those obtained from lean controls, indicating a Th1 polarization in obese AT [16, 17]. The crucial contribution of Th1 cells to adipose inflammation and metabolic dysfunctions in obesity is supported by the fact that deficiency of both CD4^+^ T cells and IFN-γ reduced adipose tissue inflammation and improved insulin sensitivity in obese ones [18]. Th1 cells and IFN-γ have been shown to deeply interfere with insulin signaling, leading to insulin resistance in adipose tissue and skeletal muscle cells, finally contributing to systemic insulin resistance in obesity [17, 19]. Here we showed that a significant proportion of CD4^+^ T cell clones derived from peripheral blood of obese patients, but not from lean metabolically healthy subjects, were activated by CD300e, proliferated, and showed a typical Th1 profile, emphasising the pathogenic role of Th1 immune dysregulation in obesity.

It is noteworthy that a predominant CD4^+^ Th1 profile was demonstrated not only in the peripheral blood but also in the adipose tissue of obese patients by CD300e-specific CD4^+^ T cells. The possibility that the anti-CD300e autoantibodies, besides the T cells specific for CD300e, might be involved in the immunopathology of obese patients deserves further investigation.

Our findings reinforce the notion that targeting specific immune system processes and metabolic pathways are crucial to control immune response and maintain immunological homeostasis, as well as to prevent (or treat) inflammation-associated metabolic changes (such as altered glucose tolerance and IR, occurring in obesity and T2D). Moreover, our results suggest that the serum anti-CD300e antibodies may become not only a useful diagnostic biomarker of the key immunometabolic perturbations leading to unhealthy obesity, but also a predictive biomarker for a successful therapeutic strategy.

## Data Availability

Data supporting the results reported in the article can be requested to the corresponding author.

## Abbreviations

IR: Insulin resistance
AT: Adipose tissue
T2D: Type 2 diabetes
HOMA-IR: Homeostatic model assessment for insulin resistance
Th: T helper
AU: Arbitrary units.
